# Undrained behavior of marine clay under one-way cyclic loading with variable confining pressure

**DOI:** 10.1038/s41598-024-63917-9

**Published:** 2024-06-12

**Authors:** Lei Sun

**Affiliations:** School of Resources and Civil Engineering, Suzhou University, Suzhou, 234000 People’s Republic of China

**Keywords:** Engineering, Materials science

## Abstract

Settlement of roads or railway caused by traffic loading has a serious effect on the safety and service performance of transport infrastructures constructed on soft marine clay. While simple cyclic triaxial test with constant confining pressure (CCP) were used in most of the previous studies, a series of cyclic triaxial tests with variable confining pressure (VCP) were carried out on normally consolidated (NC) and overconsolidated (OC) reconstituted Wenzhou soft clay in this paper to study the undrained behavior of soft marine clay due to surcharge preloading as well as cyclic traffic loading. VCP test is able to approximate the complicated stress path than CCP test caused by traffic loading. The test results indicate that NC specimens show significantly different pore water pressure (*u*) evolution compared with OC specimens. However, a change in the overconsolidation ratio (OCR) of OC specimens does not have a significant influence on variation of *u* with identical cyclic stress ratio (CSR) and total stress path (*η*). The slope of the effective stress path is dependent on the total stress path under which the specimen was loaded cyclically, regardless of the OCR and whether variable confining pressure was applied. The slope of the effective stress path is broadly in line with the slope of corresponding total stress path. In addition, the development of permanent axial strain (*ε*^*p*^_*a*_) due to one-way cyclic loading was shown to depend significantly on the values of *η* and OCR. Both the variable confining pressure and OCR limited the strain accumulation of saturated marine clay under undrained one-way cyclic loading. Finally, the effects of *η* and OCR on the magnitude of *ε*^*p*^_*a*_ after 1000 loading cycles are quantified and incorporated in a power law function for the permanent deformation prediction of soft marine clay due to traffic loading.

## Introduction

**Figure 1:**
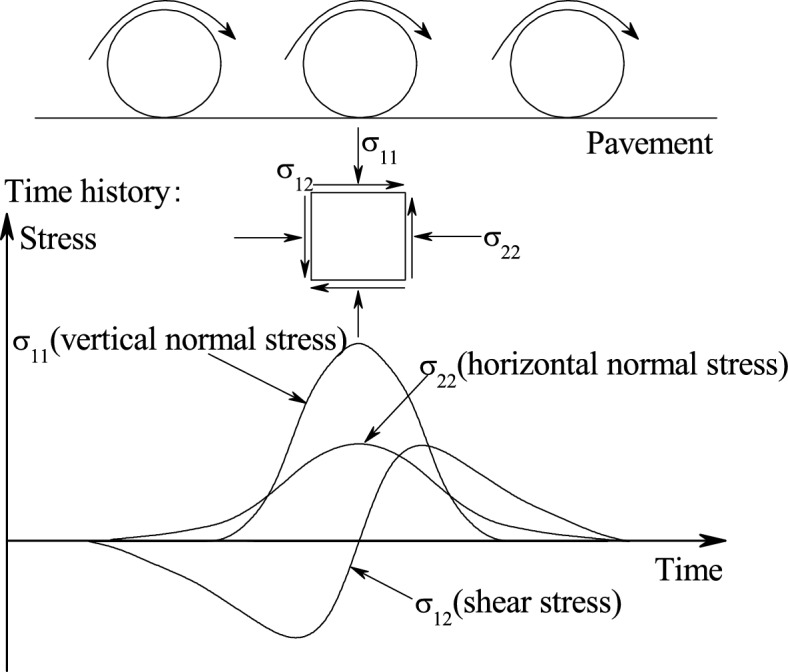
Stress variation in subsoil due to traffic load.

The deformation of soft marine clay due traffic loading leads to the settlement of traffic infrastructures such as railways, motorways and airport runways constructed on low embankments in soft marine clay area^1,2^. Excessive settlement greatly compromises the functionality and operation safety of transportation structures^3^. Therefore, knowledge of the soft marine clays’ dynamic response to traffic loading is crucial for potential settlement estimation of existing and to be constructed transport infrastructures. The soft clay’s undrained cyclic response subjected to traffic loading, generally in terms of pore water pressure and strain buildup, has been researched widely in the past by means of conventional undrained dynamic triaxial tests. Routine factors that affect the cyclic pore pressure and axial strain response, such as cyclic stress level, initial static deviatoric stress, cycle numbers (*N*) and overconsolidation ratio (OCR) have been studied extensively^4–9^. Whereas, in most of the relevance published works, traffic loading was simplified as a single one-sided (compression) cyclic axial stress with a constant confining pressure (CCP) condition. Actually, as shown in Fig. 1, the horizontal dynamic stress amplitude (σ_22_) in subgrade soil leaded by vehicle travelling is a considerable value, which should be considered with vertical dynamic stress together for a better characterization of traffic loading stress path^10^. To illustrate the combined power of vertical and horizontal dynamic stress on the cyclic response of sandy soils such as resilient moduli (*M*_*r*_), shear and volumetric strains (*ε*_*s*_, *ε*_*v*_), various test attempts through different cyclic triaxial stress path patterns have been conducted based on the drained cyclic triaxial experiment^11–14^. In these above laboratory test findings, a parameter *η* representing the slope of the stress path in* p*′-*q* plane is proposed to quantify the effect of variable confining pressure (VCP). The stress parameters *p*′ = (σ′_1_ + 2σ′_3_)/3 and *q* = (σ′_1_ − σ′_3_), where σ′_1_ and σ′_3_ are the effective vertical and lateral stresses respectively.

Experimental studies have indicated that VCP is able to decrease the dynamic strength, damping ratio and plastic strain of soft clay under undrained conditions^15–17^. Cai et al.^18^ and Sun et al.^19^ further investigated the permanent strain behavior of remolded and intact Wenzhou soft clay under VCP stress paths simulating traffic loading in drained conditions respectively, which concluded that VCP promote the development of plastic strain, plastic compressed volumetric strain under drained conditions. Actually, the test soft clay specimens are usually perceived as partially drained condition during cyclic loading due to the low permeability and remained in more qualitative analysis. During partially drained cyclic test, the cyclic response such as pore pressure, axial strain, volumetric strain and effective stress paths cannot be uniquely identified, which necessarily varied with the different degrees of drainage induced by loading patterns, frequency, soil fabric, instrument type and so on. Moreover, when the partially drained cyclic test results are used for finite element analysis, how to determine drainage boundaries reasonably is also up against a great challenge, whereas the drainage boundary effects of cyclic tests in fully drained or undrained condition can be ignored.

Up to now, owing to the limited amount of undrained VCP laboratory test results, the knowledge on the effects of VCP along with different overconsolidated ratio (OCR) factors on the important phenomenon such as excess pore water pressure, effective stress path, permanent axial strain evolution as well as empirical formulas remains limited. This paper seeks to further explore these problems through a series of undrained cyclic compression tests on reconstituted Wenzhou soft marine clay.

## Test apparatus and schemes

### Testing apparatus

**Figure 2:**
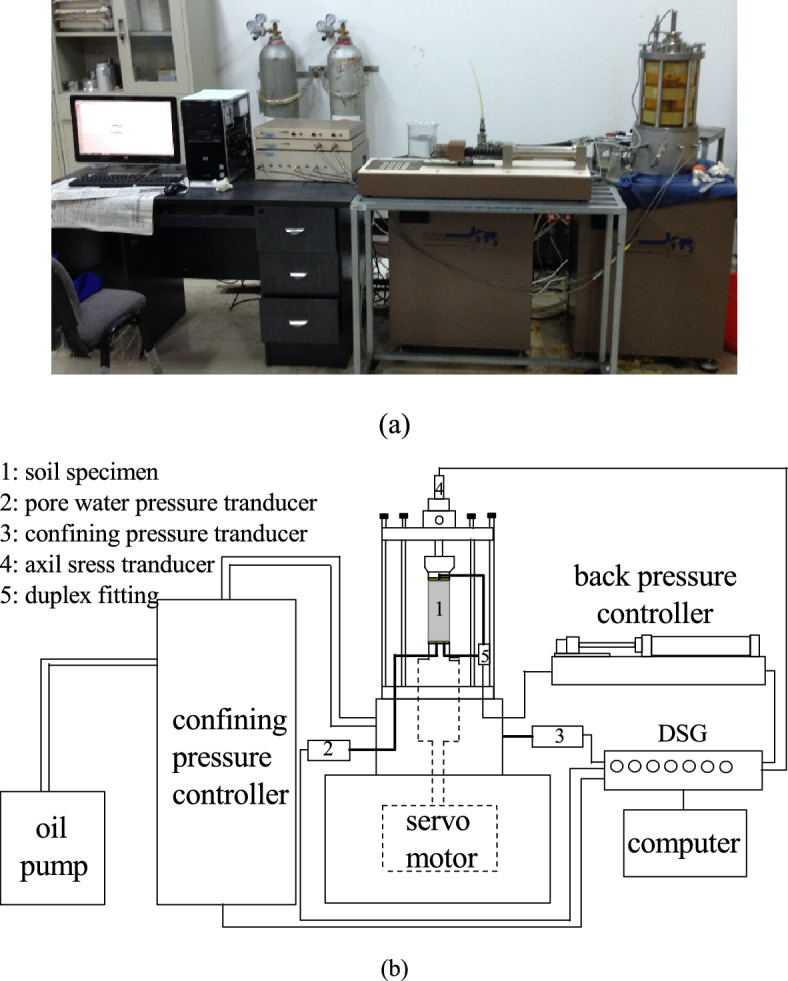
Laboratory test system (**a**) General view of GDS dynamic triaxial; (**b**) Schematic diagram of GDS dynamic triaxial.

In this paper, an advanced dynamic triaxial experimental system imported from England with high precision (Fig. 2a) was employed to develop Wenzhou soft clay’s undrained cyclic response due to traffic load. The test system can carry out both stress or strain controlled cyclic tests through such as sinusoidal semisinusoidal and customized waveform and so on. Compared to conventional dynamic triaxial, the highlight of the test system reflected in the independently controlling of axial cyclic deviator stress, *q*^*ampl*^ as well as variable confining pressure, *σ*_*3*_^*ampl*^ and so aided the traffic-induced vertical-horizontal coupling cyclic stress path simulation^15^. The platform structural schematic for a clear view of the function and linkage of each part are further plotted in Fig. 2b.

### Test soil and sample preparation

Wenzhou soft marine clay is adopted in this study, which has been widely reported for its miserable engineering properties named poor permeability, low strength, and high compressibility^2,7^. The block samples of Wenzhou soft marine clay were taken from an 8 to 10 m deep pit using a customized block sample. The basic laboratory measured soil indexes are listed in Table 1.

**Table 1:** Physical properties of Wenzhou soft marine clay.

Index properties	Values
Specific gravity, *G*_s_ (g/cm^3^)	2.71
Natural water content, *w*_n_ (%)	59–61
Initial density, *ρ*_0_ (g/cm^3^)	1.62–1.65
Initial void ratio, *e*_0_	1.59–1.62
Liquid limit, *w*_L_ (%)	60
Plasticity index, *I*_p_	35–37
Clay fraction, (%)	41
Silt fraction, (%)	55
Permeability coefficient, *k* (cm/s)	1.78 × 10^–7^

To eliminate the intact soil's structure effect on the test results and ensure the uniformity of the sample quality, All the tests in this paper conducted on remolded marine clay specimens. The standard reconstituted soil's preparation procedure suggested by Gu et al.^15^ is as follows:

- The in-situ collected marine clay was first dried in an oven at 105 °C and then made into powder.
- Mixing the distilled water into the marine clay powder to produce the slurry of marine clay with a water content of about 1.5 times the liquid limit.
- Pouring the marine clay slurry into a large oedometer and preconsolidated under a vertical pressure of 100 kPa until the vertical deformation rate was lower than 0.1 mm per day.

**Figure 3:**
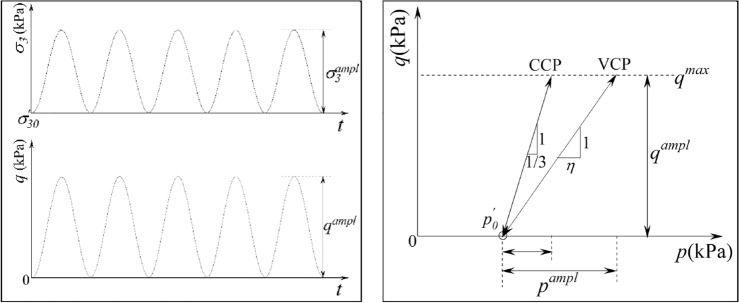
Cyclic triaxial loading waveforms and stress paths (**a**) cyclic deviator stress and variable confining pressure; (**b**) CCP and VCP stress paths.

All solid cylinder specimens were cut from the middles of preconsolidated marine clay blocks by a wire saw. After hand trimmed to normal size (D = 39.1 mm, H = 80 mm), the specimen was enclosed with a latex membrane and mounted in the pressure chamber of testing system. Subsequently, the specimen was backpressure saturated until B-values of all specimens larger than 97% and then consolidated. An effective confining pressure magnitudes (*p′*_*0*_ = 100 kPa) and five overconsolidation ratios (OCR = 1,1.5,2,3,4) were selected to mimic real initial stress conditions. It should be noted that all the overconsolidated (OCR = 1.5,2,3,4) specimens was achieved by two stages consolidation with final confining pressure value *p′*_*0*_ = 100 kPa. For example, a specimen was firstly preconsolidated at *p′*_*OC*_ = 200 kPa, and then swelling back to *p′*_*0*_ = 100 kPa, a specimen with OCR = *p′*_*OC*_/*p′*_*0*_ = 2 was achieved. Altering the value of *p′*_*OC*_ and repeating above consolidation process, the specimens with different OCRs can also be obtained. Afterward, the consolidated specimen was one-sided cyclic loaded as shown in Fig. 3a under different stress levels (*q*^*ampl*^) and stress paths (*η*) in undrained condition. According to the suggesting of Cai et al.^16^, the VCP test pattern with identical *p′*_*0*_ illustrated in Fig. 3b and 0.1 Hz loading frequency were adopted in this study. All tests were conducted with identical cyclic stress ratio (CSR) to facilitate the VCP and OCR effect analysis. Here, CSR is defined as the ratio of the cyclic deviatoric stress amplitude (*q*^*ampl*^) to the static undrained triaxial shear strength (*q*_*f*_)^16^.

1 $$ {\text{CSR}} = q^{ampl} /q_{f} $$

where, the values of *q*_*f*_ were the deviator stress at which the axial strain of soil specimen reached 15% in a consolidated-undrained (CU) triaxial test. Five CU tests were conducted at the selected OCRs of 1.0, 1.5, 2.0, 3.0 and 4.0 before cyclic triaxial tests. The preliminary CU tests showed that *q*_*f*_ are approximately 63.6 kPa, 84.6 kPa, 101.2 kPa, 123.3 kPa and 144.5 kPa for OCR = 1.0, 1.5, 2.0, 3.0 and 4.0, respectively. The detailed CCP and VCP cyclic triaxial tests scheme is listed in Table 2

**Table 2:** CCP and VCP tests scheme.

Specimen ID	*p′*_*0*_: kPa	OCR	CSR	*ƞ*
UD­01	100	1	0. 5	1/3
UD­02	100	1	0. 5	1.0
UD­03	100	1.5	0. 5	1/3
UD­04	100	1.5	0. 5	1.5
UD­05	100	2	0. 5	1/3
UD­06	100	2	0. 5	1.0
UD­07	100	2	0. 5	2
UD­08	100	3	0. 5	1/3
UD­09	100	3	0. 5	1.0
UD­10	100	3	0. 5	1.5
UD­11	100	4	0. 5	1/3
UD­12	100	4	0. 5	1.0
UD­13	100	4	0. 5	2

## Test results and analysis

### Typical CCP and VCP test results

**Figure 4:**
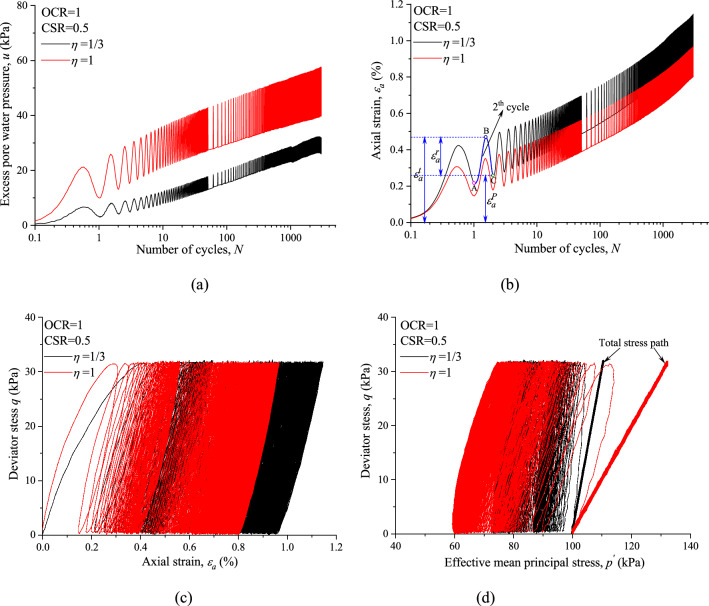
Comparisons of CCP and VCP test results for normally consolidated marine clay: (**a**) excess pore water pressure; (**b**) axial strain; (**c**) hysteretic loops; (**d**) stress path.

**Figure 5:**
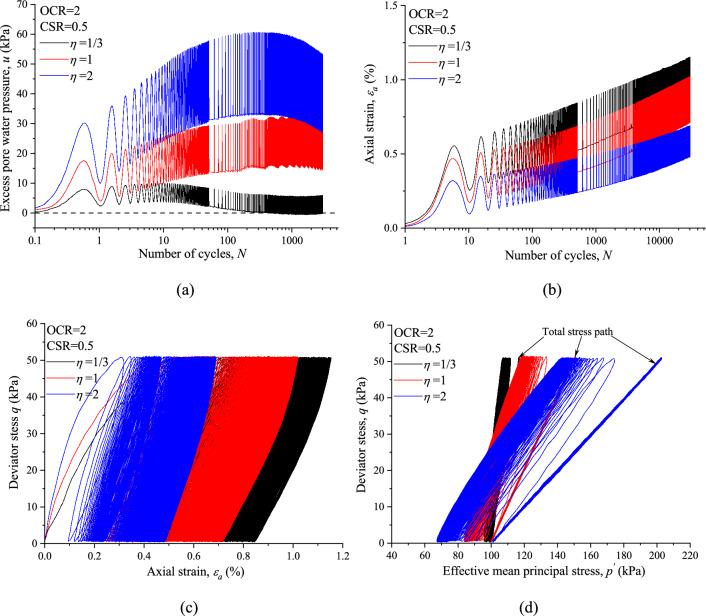
Comparisons of CCP and VCP test results for overconsolidated marine clay (OCR = 2): (**a**) excess pore water pressure; (**b**) axial strain; (**c**) hysteretic loops; (**d**) stress path.

Typical plots of CCP and VCP test results of normally consolidated (NC) and overconsolidated (OC) marine clay are shown in Figs. 4 and 5, respectably. Overall, both variable confining pressure and OCR have great influence on the undrained cyclic response of saturated marine clay. As shown in Figs. 4a and 5a, VCP tests resulted in a larger *u* with the identical number of cycles for both NC and OC specimens. Figures 4b and 5b show the development of (*ε*_*a*_) with *N* for NC and OC specimens under CCP and VCP test conditions. It can be noted from Fig. 4b that, *ε*_*a*_ can be decomposed into permanent axial strain (*ε*^*p*^_*a*_) and resilient axial strain (*ε*^*r*^_*a*_). Comparing the magnitudes of *ε*_*a*_ between CCP and VCP tests shows that higher value of *η* caused a smaller *ε*_*a*_ with the same CSR and *N*, which indicates that the build-up of axial strain in VCP test was limited due to the discipline effect of variable confining pressure.

Deviator stress is plotted against axial strain in Figs. 4c and 5c, suggest that the shapes of VCP and CCP stress strain curves are similar to each other, except for the area of the hysteretic loops. The aera of the stress strain hysteretic loop corresponds to the dissipated energy during one single cycle tends to decrease with increasing loading cycles for both CCP and VCP test. It is also observed that the energy dissipates quickly at the primary cycles, then the dissipation rate tends to decrease with increasing cycle numbers and finally reach a steady state with a constant dissipated energy in each cycle.

Figures 4d and 5d give the total stress paths for VCP and CCP tests under which the specimen was loaded cyclically as well as the measured effective stress paths, which suggests that the variable confining pressure also has a dramatic effect on the evolution trend of effective stress path due to the different variation of excess pore water pressure between VCP and CCP tests. It is also noted that the slope of effective stress path seems to in line with the corresponding slope of total stress path for both VCP and CCP test conditions.

**Figure 6:**
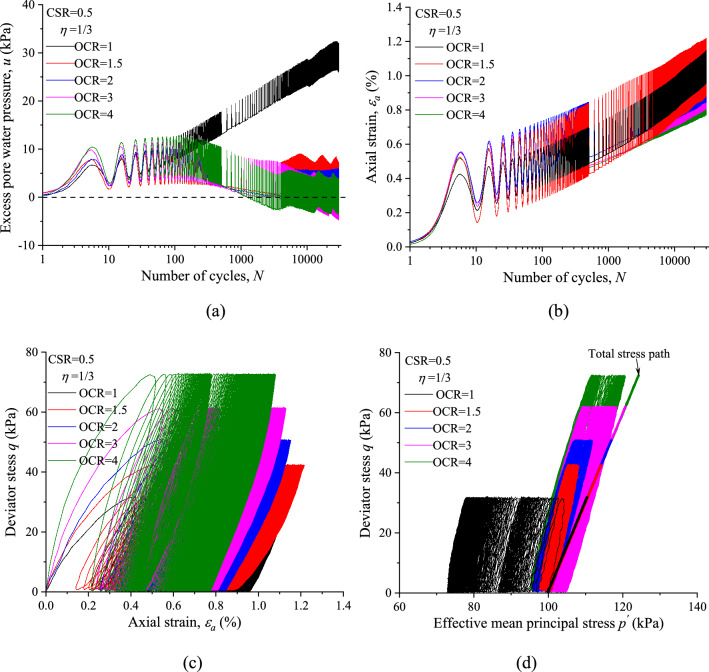
CCP test results of marine clay with different OCRs (*η* = 1/3): (**a**) excess pore water pressure; (**b**) axial strain; (**c**) hysteretic loops; (**d**) stress path.

**Figure 7:**
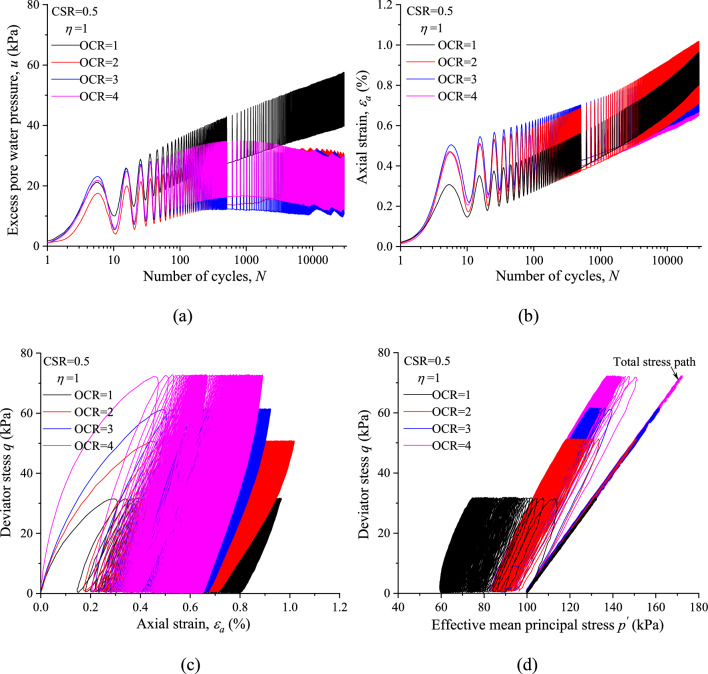
VCP test results of marine clay with different OCRs (*η* = 1): (**a**) excess pore water pressure; (**b**) axial strain; (**c**) hysteretic loops; (**d**) stress path.

To analyze the effects of stress history on the undrained cyclic response of saturated marine clay. The typical CCP test results (*η* = 1/3) and VCP test results (*η* = 1) with different OCRs are presented in Figs. 6 and 7, respectably. Figures 6a and 7a give the development of *u* over 3000 cycles in NC and OC specimens with identical CSR and *η*. Clearly, the evolution trend of *u* with *N* shows significant differences between NC and OC specimens for both CCP and VCP tests. While the curves of *u* for all the OC specimens are overlapped to each other, and presents similar evolution trend with increasing *N*. This observation suggests that the stress history plays a major role in the development of *u*.

The influence of OCR on the development of axial strain is illustrated in Figs. 6b and 7b. The results indicate that at the same CSR and *η* the permanent axial strain (*ε*^*p*^_*a*_) is consistently higher if the OCR is lower after 1000 cycles. Figures 6c and 7c compare the stress strain hysteretic loops of specimens among different OCRs but identical CSR and *η*. In general, a clear shift leftward of stress strain curve along with a reduction of the *ε*^*p*^_*a*_ with the increase of OCR may be observed.

The development of effective stress paths of NC and OC specimens with identical CSR and *η* under CCP and VCP stress paths are presented in Figs. 6d and 7d, respectably. It is evident from the figures that at the same CSR and *η* the slopes of effective stress path under different OCRs are generally similar to each other, which indicates that the slope of the effective stress path is depended on the total stress path under which the specimen was loaded cyclically, regardless of the stress history.

### Excess pore water pressure characteristic

**Figure 8:**
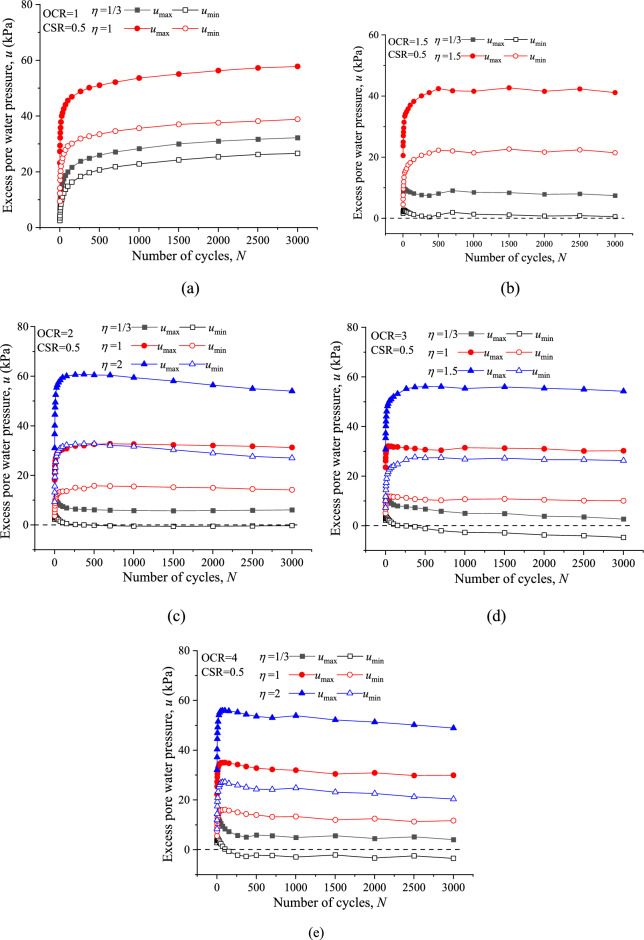
Comparisons of CCP and VCP Pore pressures response: (**a**) OCR = 1; (**b**) OCR = 1.5; (**c**) OCR = 2; (**d**) OCR = 3; (**e**) OCR = 4.

For batter view and comparison, the max and min excess pore water pressure values at selected number of cycles for all specimens are presented in Fig. 8. Qualitatively, VCP stress paths always resulted in a larger max excess pore water pressure (*u*_max_) as well as min excess pore water pressure (*u*_min_) with identical CSR and *N*, regardless of the OCRs. For example, in the case of OCR = 2 and CSR = 0.5 (shown in Fig. 8c), the *u*_max_ after 3000 cycles, with *ƞ* = 1 and 2 are 31.27 kPa and 53.99 kPa, which increase 4.15 and 7.89 times than that with *ƞ* = 1/3 (6.07 kPa). On the other hand, the evolution of *u*_max_ and *u*_min_ with the increase of *N* changes markedly due to the OCR and VCP effect. For NC specimens, both the magnitudes of *u*_max_ and *u*_min_ accumulate rapidly at the initial 1000 cycles then stabilize. While for OC specimens, the magnitudes of *u*_max_ as well as *u*_min_ accumulates quickly to a peak value at the primary stage and then decreases gradually with increasing loading cycles. The values of *u*_min_ at a larger OCR even decreased to a negative value in CCP test as shown in Fig. 8d and e. These observations suggest that the excess pore pressure evolution is strongly influenced by both the cyclic stress path (*ƞ*) and stress history (OCR), which is consistent with the results of Ningbo soft clay reported by Huang et al.^17^.

### Permanent axial strain characteristic

**Figure 9:**
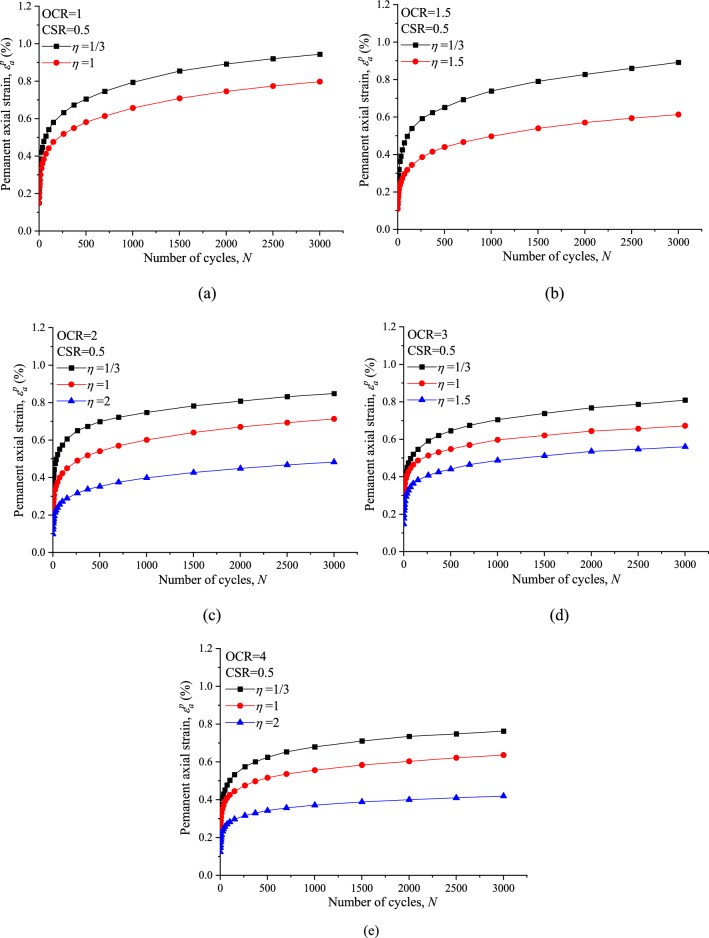
Comparisons of CCP and VCP permanent axial strain response: (**a**) OCR = 1; (**b**) OCR = 1.5; (**c**) OCR = 2; (**d**) OCR = 3; (**e**) OCR = 4.

Figure 9 illustrates the permanent axial strain (*ε*^*p*^_*a*_) of the saturated marine clay with different values of OCR and *ƞ* under the same CSR = 0.5. Generally, All the specimens deliver similar development curves of *ε*^*p*^_*a*_, which can be explained in terms of two different stages. *ε*^*p*^_*a*_ accumulates quickly at the initial stage, and then the rate of *ε*^*p*^_*a*_ development decreased and *ε*^*p*^_*a*_ accumulated at an almost constant rate under the application of subsequent cycles of loading. Under otherwise identical conditions, the cumulative *ε*^*p*^_*a*_ decreased with increasing *ƞ*.

It should be noted that variable confining pressure (VCP) causes simultaneously larger pore water pressure and smaller permanent axial strain in undrained condition. Considering that larger pore water pressure means smaller effective stress and potentially softer soil, this trend seems counterintuitive. Actually, comparing the VCP and corresponding CCP test, the deviatoric part of the stress amplitude (*q*^ampl^) was identical and held constant, larger variable confining pressure means larger isotropic amplitude (*p*^ampl^), the soil stiffness and strength depend on the effective stress, instead of pore water pressure. The reason VCP test causes smaller *ε*^*p*^_*a*_ is that the variable confining pressure restraint effect is larger than the pore water pressure softening effect.

To quantify the VCP effect on the permanent axial strain (*ε*^*p*^_*a*_), a ratio (*R*_η_) of the VCP permanent axial (*ε*^*p*^_*a,VCP*_) to the corresponding CCP permanent axial (*ε*^*p*^_*a,CCP*_) is defined as follows:

2 $$ {R_\upeta } = {\varepsilon ^p}_{a,{\text{VCP}}}/{\varepsilon ^p}_{a,{\text{CCP}}}$$

**Figure 10:**
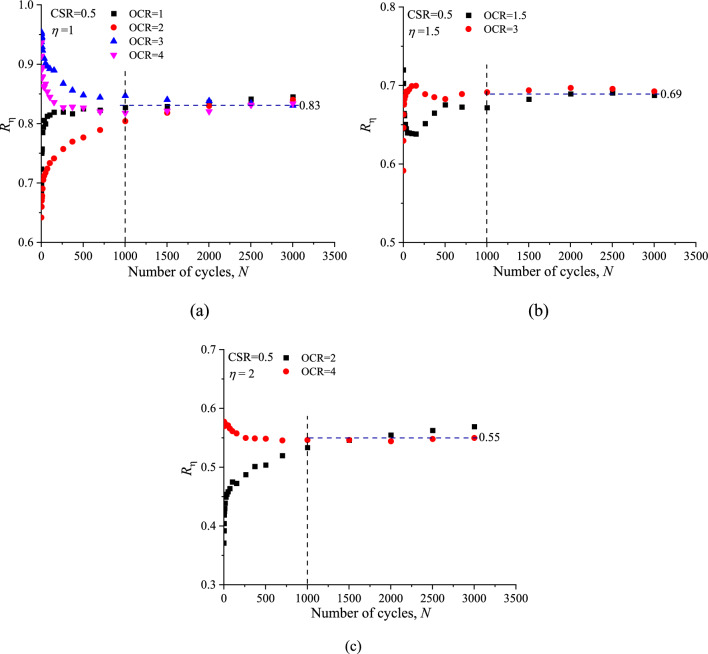
Ratio of VCP permanent axial strain to corresponding CCP permanent axial strain: (**a**) *η* = 1; (**b**) *η* = 1.5; (**c**) *η* = 2.

The variation of *R*_η_ with the same CSR and *ƞ* but different OCR values is presented in Fig. 10. The curves suggest that at the same CSR and *ƞ* values *R*_η_ fluctuated markedly before 1000 load cycles, and then tend to a constant value to the end, regardless of *N* and OCRs. As shown in Fig. 10a–c, at CSR = 0.5, the value of *R*_η_ after 1000 load cycles for *ƞ* = 1, 1.5 and 2 are 0.83, 0.69 and 0.55, respectively.

**Figure 11:**
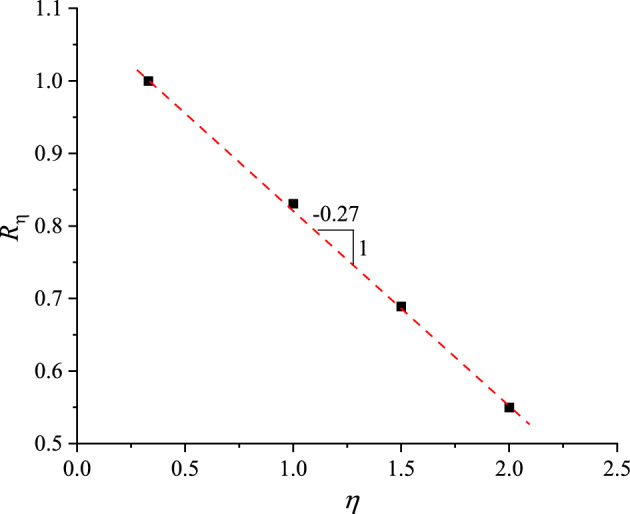
Relationship between ratio of permanent axial strain and stress path.

Figure 11 presents the relationship between *R*_1_ and *ƞ*, which can be fitted by a straight line in the following expressing:

3 $$ R_{\eta } = 0.{27}\eta + {1}.0{94} $$

Taking Eqs. (2) to (3), the prediction equation of *ε*^*p*^_*a*_ after 1000 loading cycles can thus be expressed as follows:

4 $$ \varepsilon_{a}^{p} = \varepsilon_{a,CCP}^{p} \left[ {1 - 0.27(\eta^{ampl} - 1/3)} \right] $$

**Figure 12:**
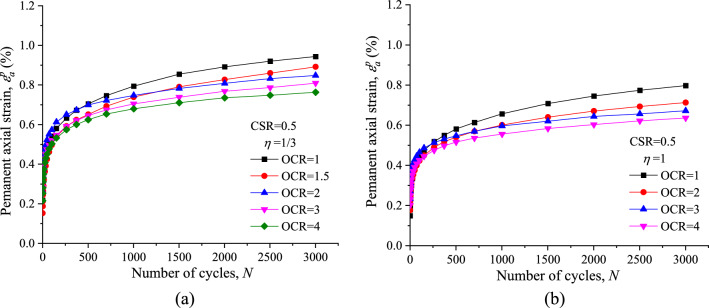
Comparisons of permanent axial strain response at different OCRs: (**a**) *η* = 1/3; (**b**) *η* = 1.

The effect of stress history on the evolution of *ε*^*p*^_*a*_ versus number of cycles is shown in Fig. 12. It can be noted that at the same CSR and *ƞ* values the pattern of *ε*^*p*^_*a*_ development curves look the same but in fact each of them is slightly different in accumulation rate with the different OCRs. At the initial 1000 cycles, the difference in the variation of *ε*^*p*^_*a*_ with *N* is relatively small and the influence of OCR is unclear. After 1000 cycles, the difference in the variation of *ε*^*p*^_*a*_ with *N* start to become apparent due to the OCR effect, and the magnitude of *ε*^*p*^_*a*_ is consistently decreased with increasing OCR.

To quantify the OCR effect on the permanent axial strain (*ε*^*p*^_*a*_), a ratio (*R*_OCR_) of the permanent axial strain for OC specimen (*ε*^*p*^_*a,*OC_) to the permanent axial strain for NC specimen (*ε*^*p*^_*a,*NC_) is defined as follows:

5 $${R_{{\text{OCR}}}} = {\varepsilon ^p}_{a,{\text{OC}}}/{\varepsilon ^p}_{a,{\text{NC}}}$$

**Figure 13:**
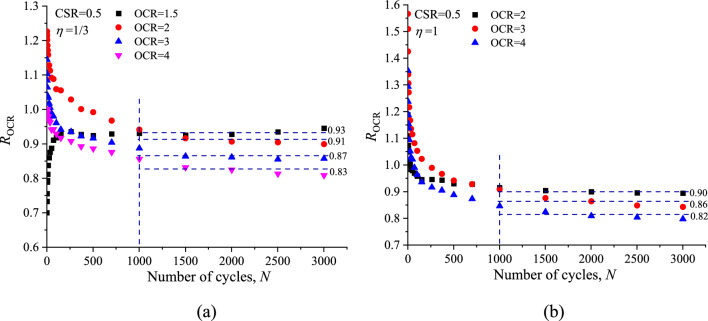
Ratio of overconsolidated specimen’s permanent axial strain to the value of normally consolidated specimen: (**a**) *η* = 1/3; (**b**) *η* = 1.

Figure 13 illustrates the variation of *R*_2_ with the identical CSR and *ƞ* but different OCR value. As can be seen that the value of *R*_OCR_ fluctuated greatly during the initial stage (*N* = 0–1000), and then tend to a constant value during the subsequent loading phase (*N* = 1000–3000).

**Figure 14:**
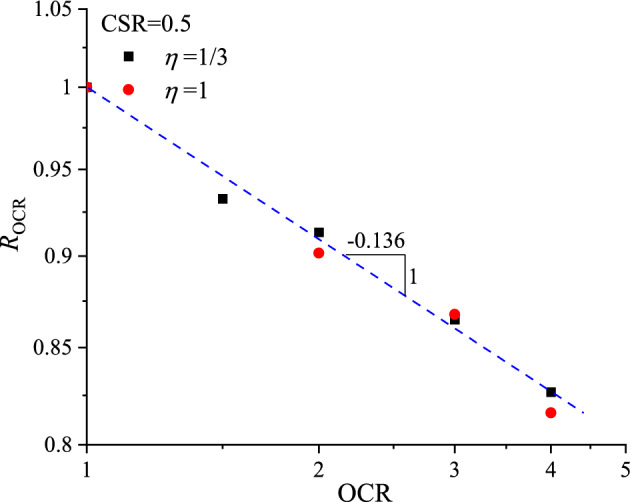
Relationship between ratio of permanent axial strain and OCR.

The values of *R*_OCR_ obtained from Fig. 13 are plotted against the OCRs of the samples in Fig. 14. In a log–log coordinate system, the values of *R*_OCR_ are linear decreased with the increasing OCR, regardless of *ƞ* values, which can be expressed as follows:

6 $$ R_{{{\text{OCR}}}} = \left( {{\text{OCR}}} \right)^{{ - 0.{136}}} $$

**Figure 15:**
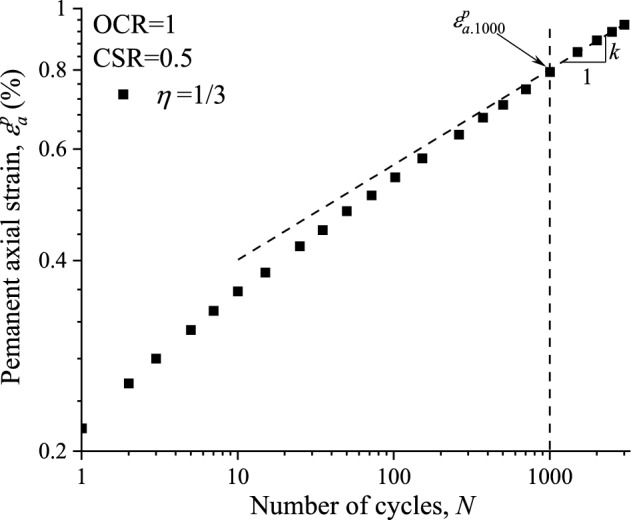
Development behavior of permanent axial strain in log–log coordinate.

According to the shakedown theory, All the accumulative curves of the permanent axial strain with loading cycles are in the regime of the stable pattern, which can be well described by a power function^16^

7 $$ \varepsilon_{a}^{p} = \varepsilon_{a,1000}^{p} \left( \frac{N}{1000} \right)^{k} $$

where *ε*^*p*^_*a,*1000_ = permanent axial strain generated in the 1000 cycle; *k* = the slope of the permanent axial strain curve in the ln*ε*^*p*^_*a*_ ˗ ln*N* coordinate after 1000 cycles. To determine the value of the parameters *ε*^*p*^_*a,*1000_ and *k*, the measured permanent axial strain of the specimen under the conditions of OCR = 1, CSR = 0.5 and *η* = 1/3 in log–log plot is illustrated Fig. 15. From regression analysis the value of the parameters can be expressed as *ε*^*p*^_*a,*1000_ = 0.794, *k* = 0.156.

Based on Eqs. (4) , (6) and (7), An equation considering the effect of cycle numbers, stress path as well as stress history is proposed to predict the permanent axial strain of saturated marine clay due to cyclic traffic loading:

8 $$ \varepsilon_{a}^{p} = 0.794\left( \frac{N}{1000} \right)^{0.156} \left[ {1 - 0.27(\eta^{ampl} - 1/3)} \right](\text{OCR})^{ - 0.136} $$

**Figure 16:**
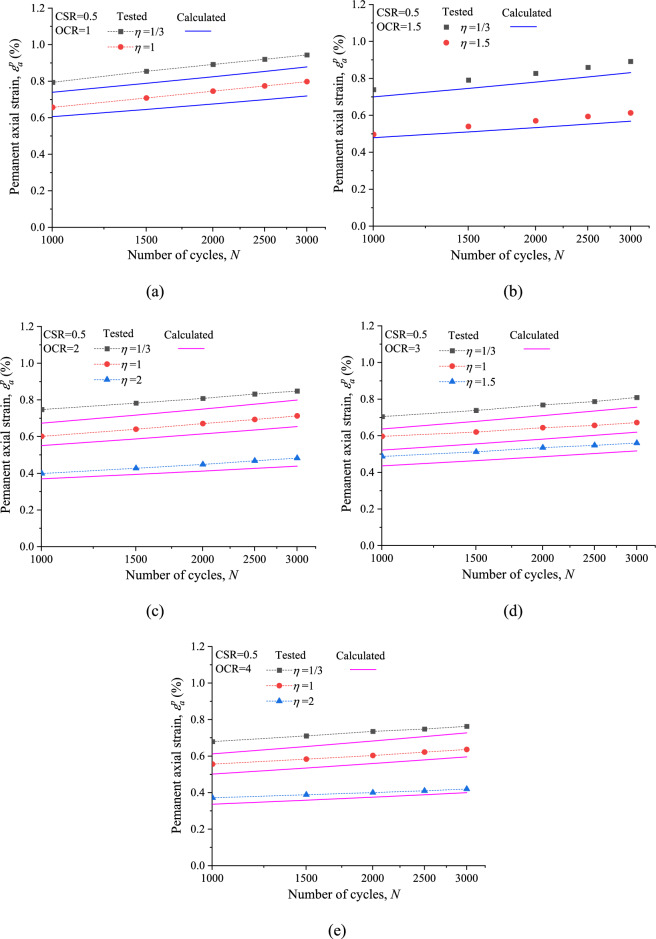
Comparison between the calculated and tested permanent axial strain: (**a**) OCR = 1; (**b**) OCR = 1.5; (**c**) OCR = 2; (**d**) OCR = 3; (**e**) OCR = 4.

The comparison between the simulated and test result of permanent axial strain is given in Fig. 16. It can be observed that the calculated curves agree well with the tested results after 1000 loading cycles, which confirms that the proposed Eq. (8) for *ε*^*p*^_*a*_ can well reflect the influences of cyclic stress path (*η*) as well as stress history (OCR) on the long-term permanent axial strain accumulation.

## Conclusions

A series of cyclic undrained triaxial tests with different stress paths (*η*) and OCRs were performed in this study to revel the pore water pressure development and strain accumulation behavior of reconstituted Wenzhou soft marine clay due to cyclic traffic loading. The major conclusions can be drawn as follows:

1. The variation of pore water pressure is strongly influenced by the variable confining pressure and OCR. Both the NC and OC specimens underwent higher pore water pressure in VCP test compared to the corresponding CCP tests. Compared to NC specimen, OC specimen presented different trends in term of pore water pressure development during undrained cyclic loading. Overconsolidation effects caused a peak in the pore water pressure curve especially in VCP tests, meanwhile, a negative pore water pressure was observed in CCP tests with larger OCRs after a certain number of cycles.
2. As a consequence of the variable confining pressure, a remarkable difference in effective stress path compared to the CCP tests was observed in VCP test. The slop of effective stress paths is broadly in line with the total stress path inclination under which the sample was loaded cyclically, regardless of the *η* and OCR.
3. All test samples showed similar trends in axial strain development as well as stress–strain hysteretic loops. At the same CSR, VCP test leads to a smaller permanent axial strain (*ε*^*p*^_*a*_) compared to the corresponding CCP tests due to the restraint effect of variable confining pressure, meanwhile, the restraint effect of variable confining pressure on the *ε*^*p*^_*a*_ is independent of the OCR after 1000 cycles. Compared to NC specimens, OC specimens underwent smaller *ε*^*p*^_*a*_ after 1000 cycles at the same CSR and *η*.
4. To get exact deformation of the marine clay due to traffic loading, the influences of cyclic stress path (*η*) and OCR should be considered. Based on the ratio of the *ε*^*p*^_*a*_ between VCP and CCP test, together with the ratio of *ε*^*p*^_*a*_ between OC and NC specimens, a power law empirical equation incorporating the effects of *η* as well as OCR is proposed, which can be used for predicting the settlement of road overlaid on soft marine clay due to traffic loading.
5. Additional test data considering the effects of CSR and Principal Stress Rotation are needed to further establish a general permanent strain empirical model for the marine subsoil in the transport infrastructures such as railways, motorways and airport runways and so on.

## Data Availability

Some data used during the study are available from the corresponding author by request.
